# Quantitative trait loci mapping for feed conversion efficiency in crucian carp (*Carassius auratus*)

**DOI:** 10.1038/s41598-017-17269-2

**Published:** 2017-12-05

**Authors:** Meixia Pang, Beide Fu, Xiaomu Yu, Haiyang Liu, Xinhua Wang, Zhan Yin, Shouqi Xie, Jingou Tong

**Affiliations:** 1 State Key Laboratory of Freshwater Ecology and Biotechnology, Institute of Hydrobiology, Chinese Academy of Sciences, Wuhan, 430072 China; 2 University of Chinese Academy of Sciences, Beijing, 100039 China

## Abstract

QTL is a chromosomal region including single gene or gene clusters that determine a quantitative trait. While feed efficiency is highly important in aquaculture fish, little genetic and genomic progresses have been made for this trait. In this study, we constructed a high-resolution genetic linkage map in a full-sib F1 family of crucian carp (*Carassius auratus*) consisting of 113 progenies with 8,460 SNP markers assigning onto 50 linkage groups (LGs). This genetic map spanned 4,047.824 cM (0.478 cM/marker) and covered 98.76% of the crucian carp genome. 35 chromosome-wide QTL affecting feed conversion efficiency (FCE, 8 QTL), relative growth rate (RGR, 9 QTL), average daily gain (ADG, 13 QTL) and average daily feed intake (ADFI, 5 QTL) were detected on 14 LGs, explaining 14.0–20.9% of the phenotypic variations. In LGs of LG16, LG25, LG36 and LG49, several QTL affecting different traits clustered together at the identical or close regions of the same linkage group. Seven candidate genes, whose biological functions may involve in the energy metabolism, digestion, biosynthesis and signal transduction, were identified from these QTL intervals by comparative genomics analysis. These results provide a basis for elucidating genetic mechanism of feed efficiency and potential marker-assisted selection in crucian carp.

## Introduction

Feed efficiency is economically important trait to animal producers because feed represents the major input in production, and the relevant discharge pollution is also a major concern^1^. It has been reported that feed accounts for 30–70% of the total costs in almost all animal production system, such as poultry industry^2^, livestock production^3,4^ and aquaculture industry^5,6^. Improvement in feed efficiency can not only reduce stocking expenses but also save rearing period, therefore enhancing economic efficiency. In addition, improvement in feed efficiency indicates benefits in environmental sustainability^7^. The Food and Agriculture Organization (FAO) of the United Nations estimated that global livestock were responsible for 18 percent of greenhouse gas emissions, including methane and nitrous oxide^8^, and evidences have shown that improvement of feed efficiency minimized the methane production in beef cattle^9,10^.

Most economic traits in animal production such as growth and disease resistance are controlled by a series of genes, external environmental factors and their interactions. Feed efficiency is also such a major trait and hard to be improved by those traditional methods such as inbreeding, selection, crossbreeding and hybridization when involving in production. It has been confirmed that feed efficiency is a heritable trait in beef cattle^11,12^, pigs^13^, chickens^14^, turkeys (*Meleagris gallopavo*)^15^, ducks^16^ and fish^17^. Thus the purpose of improvement in feed efficiency could be achieved by selecting animals that are genetically superior. Quantitative trait loci (QTL) mapping is a supplementary means to assist the selection of desired traits^18,19^ because a QTL is a chromosomal region including single gene or gene clusters that determine a quantitative trait^20^. Now QTL mapping for economically important traits is an essential means for marker-assisted selection (MAS) in fish breeding programs. As a matter of fact, the genetic bases for performance traits, such as growth in rainbow trout (*Oncorhynchus mykiss*)^21^ and common carp (*Cyprinus carpio*)^22^; hypoxia tolerance in channel catfish^23^; sexual maturation in Arctic charr (*Salvelinus alpinus*)^24,25^ and rainbow trout^26^; disease resistance in Atlantic salmon (*Salmo salar*)^27^ and Japanese flounder (*Paralichthys olivaceus*)^28^ have been well studied through QTL mapping.

Selection to improve feed efficiency, which usually measured as feed conversion ratio (FCR: the ratio of feed to gain) or residual feed intake (RFI: the actual feed intake minus the predicted feed intake based on growth and body weight of an animal^29^), has the potential to increase growth rate and cut production costs in young animals because these two traits genetically correlated^30^. Progresses to identify genetic markers involved in feed efficiency have been made especially in livestock, which focused on cattle and pigs by genome association studies^31,32^ and QTL mapping using microsatellite^33^ or SNP markers^34^. Some candidate QTL related to feed efficiency have also been made public in poultry^35–38^ through association and linkage analyses.

It is obviously that improvements for the efficiency of feed utilization would also lead to increasing the producer’s profitability in aquaculture, however, genetic studies on feed efficiency have received less attention than other economic traits in fish production system because of the difficulties in obtaining phenotype data. First, feed intake of each fish is generally difficult to measure because the shifty feed intake over days and the feeds are hard to recycle. Second, the requirement of a set of single tank to raise individual fish in each of the reference families is generally difficult to achieve. To date, few QTL analyses associating with feed efficiency have been reported in aquaculture species^17,39^. Nevertheless, due to financial returns strongly influenced by feed efficiency, this trait needs further attention and studies in more aquaculture species^39^.

Crucian carp (*Carassius auratus*) is a member of the family Cyprinidae, which is cultivated in freshwater systems all over the world^40^. Crucian carp has a large production that increased from 2.0 million tons in 1950 to 19.6 million tons in 2014 worldwide (FAO), this means that the input costs are heavy burden for farmers, especially feed cost. The existing progresses about improving feed efficiency focused on external condition aspects, such as changing feed ingredients^41^ and different feeding ways^42^. No analysis had focused on internal molecular genetic aspects in crucian carp.

In the present study, a linkage map with 8,460 SNP markers was constructed using 2b-RAD technology^43^ in a full-sib family of diploid crucian carp. We aimed to identify QTL intervals related to feed conversion efficiency (FCE, the inverse of FCR), relative growth rate (RGR), average daily gain (ADG) and average daily feed intake (ADFI) using this high-density linkage map. Furthermore, some potential candidate genes were predicted from QTL regions by comparative genomics to provide information for elucidating genetic mechanisms underlying feed efficiency. Our analysis would lay a foundation for genetically improving feed efficiency of crucian carp in future.

## Results

### Phenotypic data

**Figure 1:**
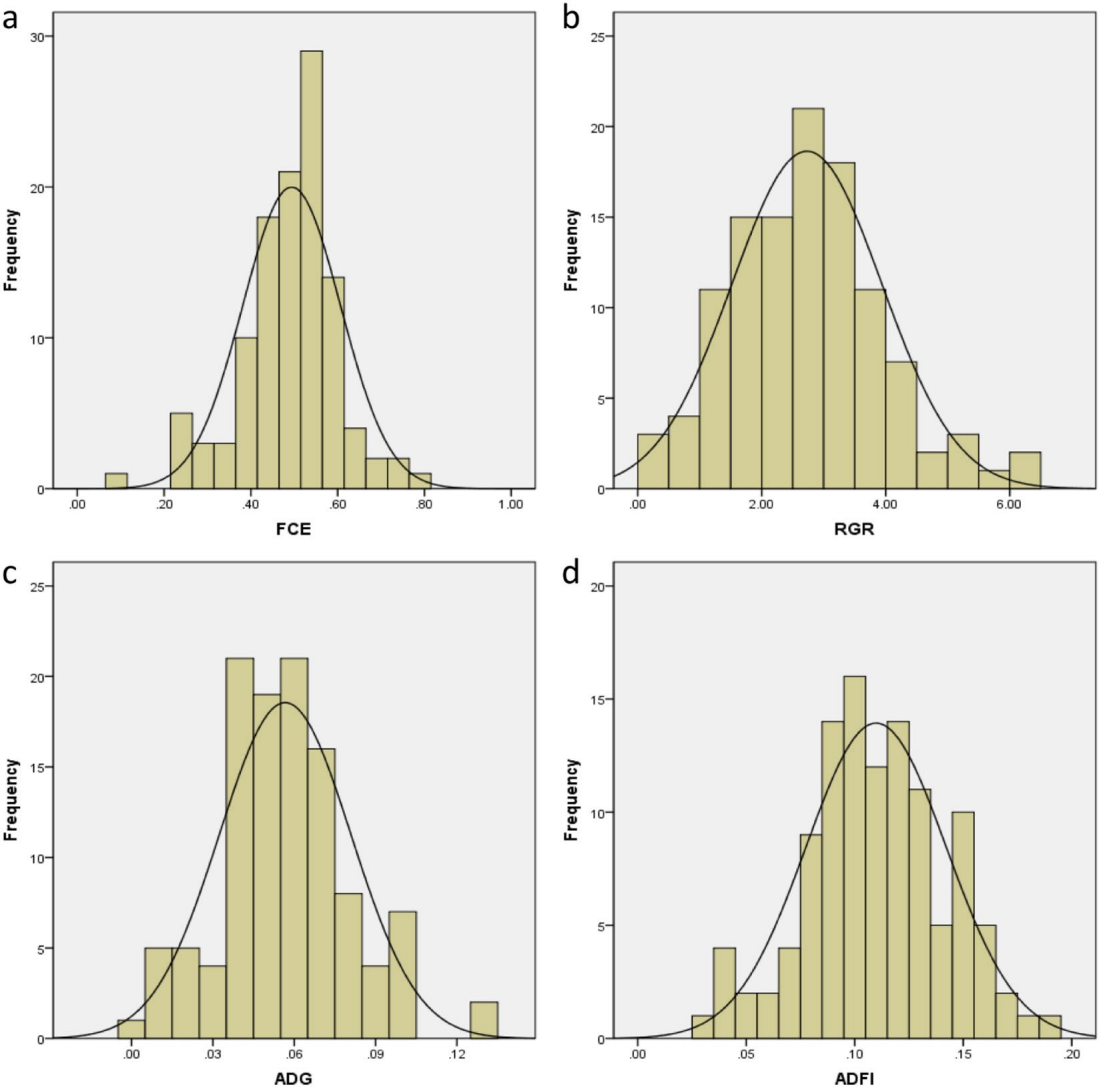
Distributions and variations of (**a**) feed conversion efficiency (FCE), (**b**) relative growth rate (RGR), (**c**) average daily gain (ADG) and (**d**) average daily feed intake (ADFI).

Out of 120 fish fed in individual tanks each, 113 were alive throughout the two-month experiment and used for the further phenotype analysis of feed efficiency. The average values of initial body weight (BW_I_) and final body weight (BW_F_) were 0.87 ± 0.39 g and 3.10 ± 1.20 g, respectively. The deduced FCE were between 9.2%-78.8% with an average value of 49.4% (SD = 11.2%), and RGR ranged from 0.19 to 6.16 with an average value of 2.73 (SD = 1.20). The average values of ADG and ADFI were 0.06 ± 0.02 g and 0.11 ± 0.03 g, respectively. All of these phenotype metrics roughly conformed to a normal distribution (Fig. 1). The relationship details among these six traits were shown in Table 1. FCE was strongly correlated with ADG (r = 0.809, *p* < *0.001*), followed by BW_F_ (r = 0.714, *p* < *0.001*), RGR (r = 0.604, *p* < *0.001*), and ADFI (r = 0.590, *p* < *0.001*) but weakly correlated with BW_I_ (r = 0.151, *p > 0.05*). BW_F_ was also highly correlated with ADG (r = 0.939, *p* < *0.001*) and ADFI (r = 0.908, *p* < *0.001*), and slightly correlated with RGR (r = 0.269, *p* < *0.05*). The Pearson correlation between ADG and ADFI (r = 0.922, *p* < *0.001*) was also significant.

**Table 1:** Pearson correlation coefficients (r) for all pairwise combinations among six traits in this study.

	FCE	BW_I_	BW_F_	RGR	ADG	ADFI
FCE						
BW_I_	0.151					
BW_F_	0.714	0.665				
RGR	0.604	−0.46	0.269			
ADG	0.809	0.387	0.939	0.526		
ADFI	0.590	0.441	0.908	0.436	0.922	

FCE: feed conversion efficiency, BW_I_/BW_F_: initial/final body weight, RGR: relative growth rate, ADG: average daily gain, ADFI: average daily feed intake.

**Figure 2:**
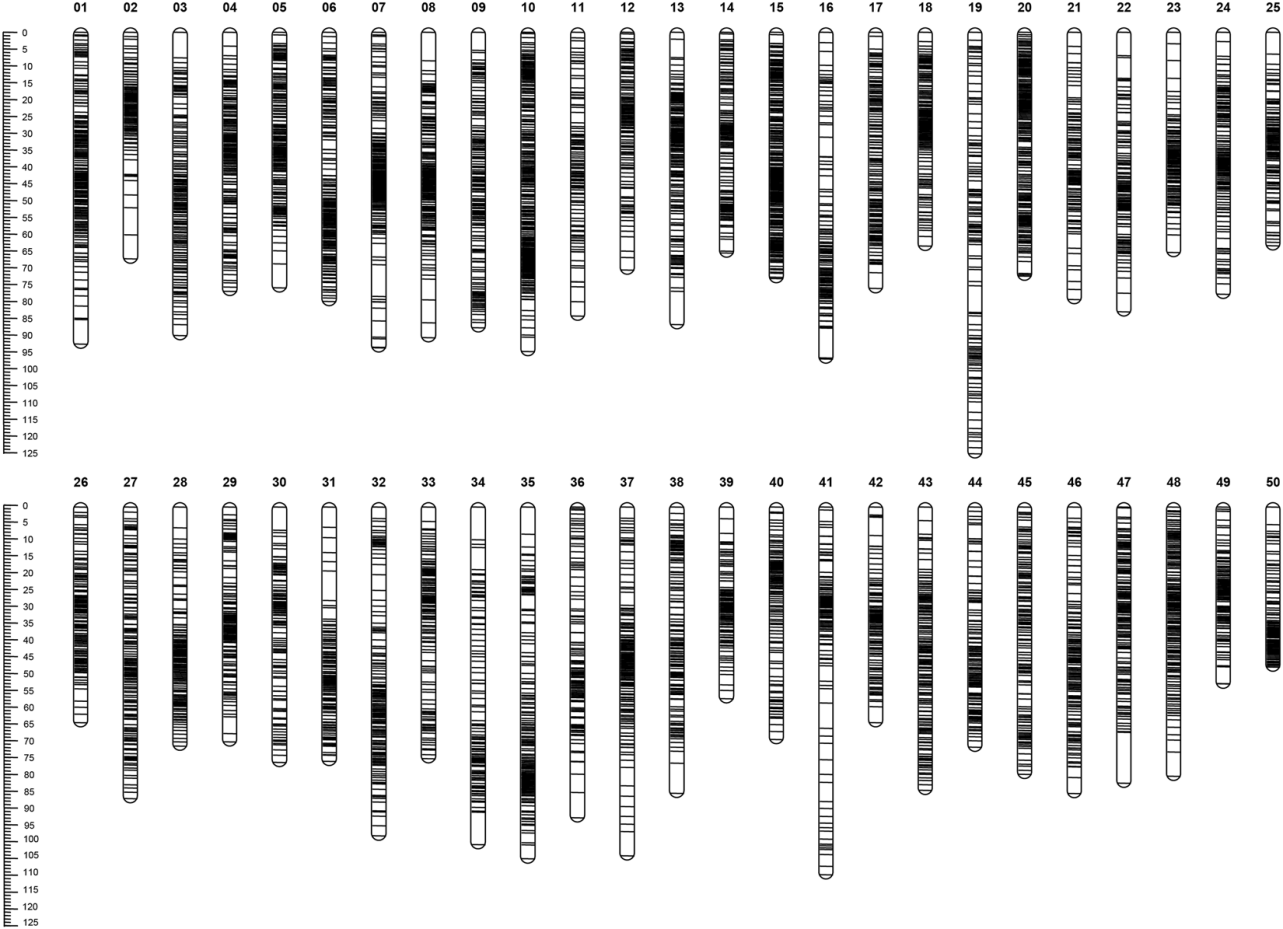
Genetic lengths and marker distribution on 50 LGs in the linkage map of crucian carp.

2b-rad genotying and genetic map construction. After filtering, a total of 173.76 million reads were produced by single-end sequencing of the mixed 2b-RAD library, which is composed of 6,120,537 reads from the female parent, 3,818,836 reads from the male parent, and 163,824,997 reads from the 113 progenies with an average of 1,424,565 reads per individual. Given that 98,336 enzyme digestion sites found in crucian carp genome, the coverage for male parent, female parent and progenies are 38.86×, 62.24× and 14.48× measured at these digestive enzyme sites, respectively. A total of 10,656 SNP loci, which were polymorphic and genotyped in at least 80% of the offspring, were used in the consecutive construction of genetic linkage map. The consensus linkage map was constructed at the logarithm of odds (LOD) threshold of 11.5 using JoinMap 4.1 software^44^, consisting of 8,460 SNP markers (Supplementary Table S1) grouped into 50 LGs (Fig. 2). The LGs of this linkage map were consistently named with those of a recently published map for crucian carp in our laboratory^45^ after a synteny analysis. This new genetic map spanned 4,047.824 cM, with the genetic distance of individual LG ranging from 56.952 cM (LG39) to 125.339 cM (LG19). The number of SNP markers varied from 118 (LG34 and LG39) to 347 (LG10) (mean 169.2), with an average interval of 0.478 cM between markers. The expected genome length of crucian carp was estimated to 4,098.707 cM, which was the average of 4,098.538 cM (G_e1_) and 4,098.875 cM (G_e2_) based on two different calculation methods^46,47^. Therefore, this present genetic map covered 98.76% of the crucian carp genome. Detailed information and characteristics of this high-density genetic map were summarized in Table 2.

**Table 2:** Summary of genetic linkage map for crucian carp.

Linkage group	Number of markers	LG length (cM)	Average marker interval (cM)
LG1	216	92.689	0.429
LG2	122	67.382	0.552
LG3	184	90.223	0.490
LG4	210	77.042	0.367
LG5	206	76.012	0.369
LG6	237	80.047	0.338
LG7	243	93.754	0.386
LG8	205	90.782	0.443
LG9	181	87.933	0.486
LG10	347	94.931	0.274
LG11	124	84.402	0.681
LG12	141	70.730	0.502
LG13	212	86.871	0.410
LG14	158	65.587	0.415
LG15	328	73.176	0.223
LG16	136	97.074	0.714
LG17	178	76.189	0.428
LG18	183	63.552	0.347
LG19	127	125.339	0.987
LG20	265	72.487	0.274
LG21	135	79.502	0.589
LG22	141	83.136	0.590
LG23	130	65.531	0.504
LG24	182	77.863	0.428
LG25	136	63.508	0.467
LG26	141	64.145	0.455
LG27	170	86.711	0.510
LG28	151	71.075	0.471
LG29	143	69.806	0.488
LG30	123	75.913	0.617
LG31	136	75.608	0.556
LG32	175	97.758	0.559
LG33	140	74.795	0.534
LG34	118	100.361	0.851
LG35	196	104.597	0.534
LG36	121	92.449	0.764
LG37	170	103.716	0.610
LG38	144	85.087	0.591
LG39	118	56.952	0.483
LG40	130	69.102	0.532
LG41	129	109.256	0.847
LG42	137	64.107	0.468
LG43	181	84.231	0.465
LG44	144	71.376	0.496
LG45	149	79.510	0.534
LG46	160	85.231	0.533
LG47	167	82.075	0.491
LG48	195	80.028	0.410
LG49	121	52.623	0.435
LG50	174	75.570	0.434
Total	8,460	4,047.824	0.478

### QTL mapping for feed efficiency

**Figure 3:**
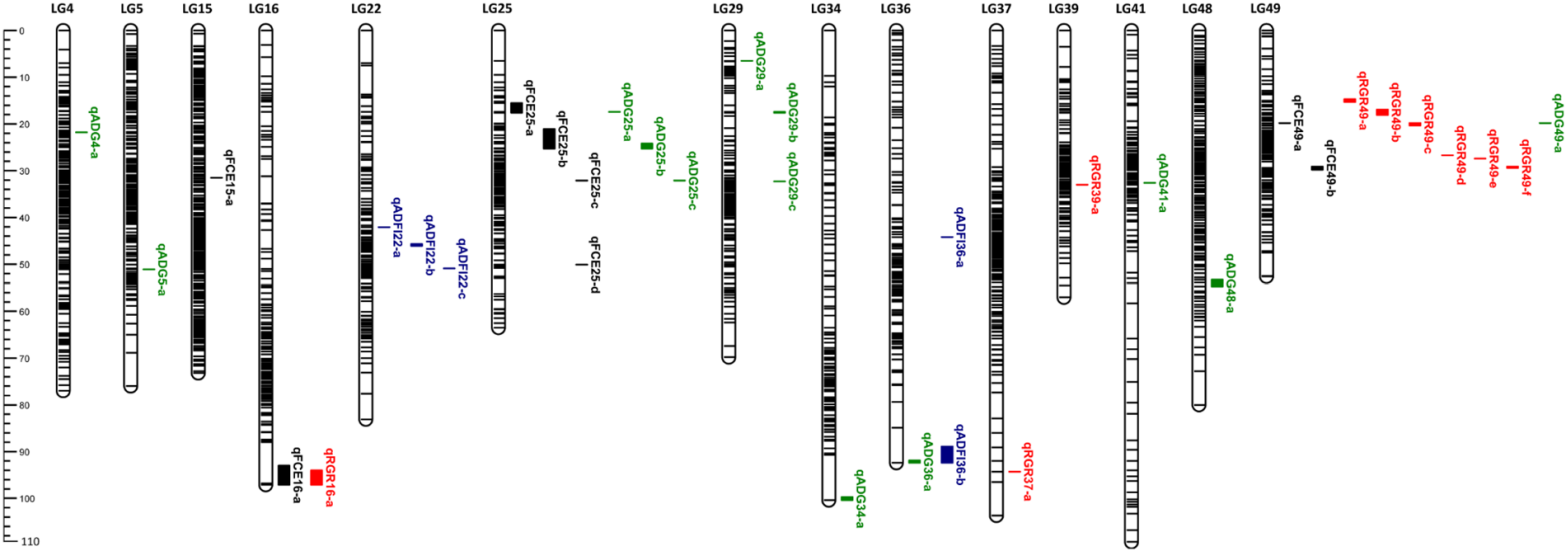
Thirty-five QTLs for FCE, RGR, ADG and ADFI distributed on 14 different linkage groups. Blank regions represent QTL for FCE, red regions represent QTL for RGR, green regions represent QTL for ADG and blue regions represent QTL for ADFI.

**Figure 4:**
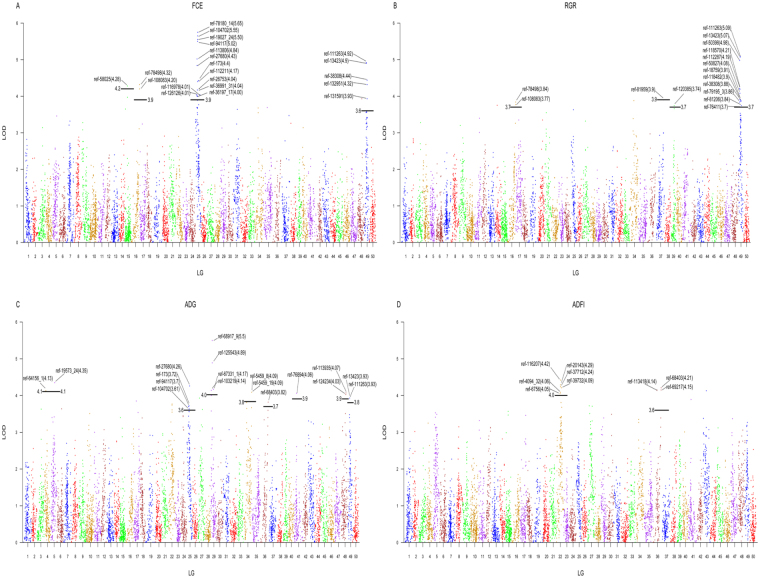
A genome scan of LOD profiles for FCE, RGR, ADG and ADFI. The solid lines indicate the chromosome-wide significance thresholds. Values in the brackets indicate the LOD thresholds of the markers. Color dots above lines represent markers associating with (**A**) FCE, (**B**) RGR, (**C**) ADG and (**D**) ADFI.

The profiles and characteristics of QTL associating with FCE, RGR, ADG and ADFI were presented in Table 3 and Fig. 3. Totally, 35 significant QTL regions were mapped onto 14 LGs using multiple QTL model (MQM) in MapQTL 6.0 program^48^ (Table 3, Figs 3, 4). The genome-wide LOD significance thresholds for the four traits ranged from 5.8 to 6.1, while the chromosome-wide LOD significance thresholds varied from 3.6 to 4.2. No QTL were detected above genome-wide thresholds for all traits. Eight QTL for FCE, which associated with 21 SNP makers in total, were found on 4 different LGs, having an effect of 15.2%-20.9% phenotypic variance explained (PVE). Nine QTL for RGR, explaining 14.1% (qRGR39-a) to 18.7% (qRGR49-c) of the phenotypic variations, were mapped onto 4 different LGs. Thirteen QTL affecting ADG were detected on 9 LGs with the PVE ranging from 14.0% (qADG25-a) to 20.1% (qADG29-b), and five QTL related to ADFI were identified on 2 LGs, accounting for 15.2–16.5% phenotypic variance (Table 3). As it shown in Fig. 3, several QTL affecting different traits clustered together at the identical or close regions of the same linkage group (LG16, LG25, LG36 and LG49), while other QTL regions were scatteredly distributed.

**Table 3:** Summary statistics of the QTL for the traits of FCE, RGR, ADG and ADFI in crucian carp.

Trait	LG	LOD threshold		QTL name	Position (cM)	No. of SNPs	LOD	Nearest marker	PVE (%)	Candidate genes
		GW	CW							
FCE	15	6.1	4.2	qFCE15-a	31.530	1	4.28	ref-58025	16.0	
	16		3.9	qFCE16-a	93.018–97.074	2	5.02	ref-78498	18.5	
	25		3.9	qFCE25-a	15.470–17.593	5	5.02	ref-94117	18.5	*mapk11*
				qFCE25-b	21.118–25.275	6	5.75	ref-78180_14	20.9	*cse1l*, *fam126b*
				qFCE25-c	32.092	1	4.43	ref-27680	16.5	
				qFCE25-d	50.082	1	4.04	ref-36991_31	15.2	
	49		3.6	qFCE49-a	19.799–19.806	2	4.92	ref-111263	18.2	*myh14*
				qFCE49-b	29.105–29.761	3	4.44	ref-38308	16.5	
RGR	16	6.0	3.7	qRGR16-a	94.018–97.074	2	4.32	ref-78498	16.2	
	37		3.9	qRGR37-a	94.339	1	3.90	ref-81959	14.7	
	39		3.7	qRGR39-a	32.986	1	3.74	ref-120385	14.1	
	49		3.7	qRGR49-a	14.675–15.285	2	4.98	ref-50398	18.4	*rgs9bp*
				qRGR49-b	16.899–18.113	4	4.19	ref-112287	15.7	
				qRGR49-c	19.799–20.341	3	5.09	ref-111263	18.7	
				qRGR49-d	26.734	1	3.84	ref-81206	14.5	*cldn10b*, *cldn10a*
				qRGR49-e	27.357	1	3.86	ref-79195_3	14.5	
				qRGR49-f	29.105–29.392	1	3.88	ref-38308	14.6	
ADG	4	5.8	4.1	qADG4-a	21.757	1	4.13	ref-64156_1	15.5	
	5		4.1	qADG5-a	51.123	1	4.35	ref-19573_24	16.2	
	25		3.6	qADG25-a	17.395	1	3.70	ref-94117	14.0	
				qADG25-b	24.229–25.275	2	3.72	ref-173	14.1	
				qADG25-c	32.092	1	4.26	ref-27680	15.9	
	29		4.0	qADG29-a	6.461–6.534	1	4.14	ref-103219	15.5	
				qADG29-b	17.414–17.636	2	5.50	ref-68917_9	20.1	
				qADG29-c	32.344	1	4.17	ref-67331_1	15.6	
	34		3.8	qADG34-a	99.686–100.361	2	4.09	ref-5459_8	15.4	
	36		3.7	qADG36-a	91.925–92.449	1	3.82	ref-68403	14.4	
	41		3.9	qADG41-a	32.621	1	4.06	ref-76894	15.3	
	48		3.9	qADG48-a	53.316–54.721	2	4.07	ref-113935	15.3	
	49		3.8	qADG49-a	19.799–19.806	2	3.93	ref-13423	14.8	
ADFI	22	5.8	4.0	qADFI22-a	42.103	1	4.05	ref-6756	15.2	
				qADFI22-b	45.667–46.053	4	4.42	ref-116207	16.5	
				qADFI22-c	50.86	1	4.06	ref-4094_32	15.3	
	36		3.6	qADFI36-a	44.17–44.173	2	4.15	ref-69217	15.5	
				qADFI36-b	88.925–92.449	1	4.21	ref-68403	15.8	

GW: genome-wide, CW: chromosome-wide, PVE: phenotypic variance explained.

### Potential candidate genes for feed efficiency

SNPs within all of the QTL intervals in this study were blasted against the assembled genome of crucian carp (Jingou Tong *et al*., unpublished data) to extend the flanking sequences of 2b-RAD markers, then those extended sequences (Supplementary Table S2) were used to blast the genome of zebrafish *D. rerio*, a species also in the family Cyprinidae, for identifying potential functional genes. Seven paralogs of the candidate genes within or close to the associated regions were identified (Table 3), including genes encoding Ran GTPase binding protein (*cse1l*), ATP binding protein (*mapk11* and *myh14*), regulator of G-protein signaling (*rgs9bp*), tight junction protein (*cldn10a* and *cldn10b*), and gene with unknown functions (*fam126b*). These potential QTL-related genes may involve in the genetic control of feed efficiency in crucian carp in such pathways as energy metabolism and signal transduction. However, their functional mechanisms and potential significance in marker (gene)-assisted selection are worthy of further studies and validation.

## Discussion

Genetic linkage maps are essential for quantitative trait loci (QTL) mapping for marker-assisted selection (MAS)^49^, and this is mainly due to the fact that high-resolution genetic linkage is one of the best tools for fine QTL mapping^50^. In this study, we constructed a high-density linkage map containing 8,460 SNP markers grouped into 50 LGs (Supplementary Table S1, Fig. 2) using 2b-RAD technology, which is in agreement with the haploid chromosome number of crucian carp^51^. The genetic map covered 98.76% of the genome with a density of 0.478 cM/marker (Table 2), demonstrating its power to detect potential QTL associating with FCE and its relative traits in crucian carp at a fine scale^34^. In order to enable the fish behave similarly compared with a production environment where they grow within a cohort, we reared the reference family fish in a single tank and let them be adapted to the fish-feeder’s behaviors for a month before the feed conversion experiment. While in order to measure the feed consumption of each fish precisely, we fed the investigated fish individually within isolated environment during the feed conversion trail. The conditions of each aquarium were regularly maintained throughout the experiment to eliminate the errors caused by environment factors as far as possible. Supposed we fed all fish in a group and estimated their feed intake with an average feed intake individually, it would have bigger errors affecting the feed efficiency metrics, because the real situation is that a wide range of variations in feed intake exist among fish used in this kind of study, even though the fish have very similar initial body weights. Finally, the deduced phenotype metrics of FCE, RGR, ADG and ADFI in this study roughly conformed to a normal distribution (Fig. 1), which reflected the real situation of fish in production conditions to a large extent.

It is well known that feed efficiency is economically important trait in most cultured species, as animals with better feed efficiency increase financial returns. Studies for finding genetic mechanisms affecting feed efficiency in livestock and poultry have been reported using different methods. For instance, Sherman *et al*. (2009) identified 19 QTL for RFI and 12 QTL for FCR in beef cattle using high-density markers on 24 autosomes^34^. Do *et al*. (2014) performed a genome-wide association analysis and identified several genes as putative candidates for RFI in pigs^32^. Yi *et al*. (2015) identified 41 differentially expressed genes associating with RFI with a correct rate of 90% by validating using RNA-Seq in chicken^52^. To date, QTL analyses involved in feed efficiency in fish are rarely public. A few QTL associating with FCR traits have been identified using SSR and EST markers in common carp^53–55^, then QTL for FCR were reported based on two mapping panels (mirror carp and hybrid carp)^17^. In the present investigation, a total of 35 QTL intervals were mapped on 14 LGs in crucian carp, including eight for FCE, nine for RGR, thirteen for ADG and five for ADFI traits (Table 3, Figs 3, 4), explainning 14.0–20.9% of the phenotypic variations (Table 3). The QTL located on different LGs and locations for the same traits revealed that FCE and its relative traits were driven by multiple loci with potentially multiple regulatory pathways^56^. Fish in different growth stages might be influenced by different sets of QTL or genomic regions, which have been observed in other fish such as rainbow trout^57^, Atlantic salmon^58^ and Asian seabass^59^. Crucian carp is a freshwater fish that sexual mature at one year old, and produced from different water systems all year round after several months to one year culturing in China. In this investigation, we designed the feed conversion trail experiment using fingerlings of crucian carp aged nearly 3 months at the beginning, which could be judged as early growth stage. The QTL obtained in this study for FCE and its relative traits may have positive significance in aquaculture and genetic selection. While the regulation mechanisms of these traits in late growth stages of crucian carp need further studies. Nevertheless, this is, to the best of our knowledge, the first QTL mapping for feed efficiency in crucian carp, an important aquaculture fish around the world.

Fish genetically superior in FCE means relatively faster growth rate and lower feed intake, thus fish selected by FCE in breeding programs can not only grow quickly but also save feed cost. In this investigation, FCE has a stronger correlation with BW_F_ (a cumulative result of body gain, r = 0.714, *p* < *0.001*) than BW_I_ (r = 0.151, *p > 0.05*) even though a noncomplete-linear correlation between BW_I_ and BW_F_ (Table 1) exists. The weak correlation between FCE and BW_I_ suggested that the body size at the beginning of reference fish individuals would not influence feed efficiency in this experiment. However, FCE was highly correlated with BW_F_ indicating that FCE is genetically highly correlated with growth. Other evidences also supported this conclusion (r = 0.809 between FCE and ADG, and r = 0.604 between FCE and RGR, all *p* < *0.001*). Although feed intake may also increase when improving feed efficiency in crucian carp of this study (r = 0.590 between FCE and ADFI, *p* < *0.001*), the correlation level was inferior to growth. While growth is also influenced by feed intake as ADG is strongly correlated with ADFI (r = 0.922, *p* < *0.001*), and both ADG and ADFI have stronger correlation with BW_F_ than BW_I_ (Table 1). Similar results have been reported in other species, for instance, body weight gain was significantly related to mean feed intake (r = 0.64, *p* < *0.001*) in channel catfish^60^ and daily dry matter intake (DMI) was highly correlated with RFI from both phenotypic and genetic aspects in beef cattle^61^.

In crucian carp, several QTL associating different traits were gathered in overlapping or nearby areas of the same linkage group, especially LG25 and LG49. For instance, as it shown in Fig. 3, QTL intervals for ADG on LG25 and LG49 were partially overlapped with those for FCE, and qADFI36-b for ADFI and qADG36-a for ADG were partially co-localized on LG36. All these genomic regions shared by different QTL provided a strong evidence for the positive correlation among different traits. Nevertheless, although most of these traits were strongly correlated, they do not share all of the same QTL locations. Similar phenomena among FCE and its relative traits have also been reported in other farmed animal species. For instance, QTL for different types of feed efficiency traits that co-localize at the same position were mapped to autosomes 16, 19, and 26 in chicken^35^. Sherman *et al*. (2009) found that some chromosomes contained QTL for both FCR and RFI, while some chromosomes contained QTL for only RFI, but all DMI QTL were on chromosomes where RFI QTL were detected in beef cattle^34^.

Feed conversion efficiency is a complex trait that involves in many physiological processes, such as feed intake, metabolism, digestion, biosynthesis, oxidative stress response and so on, which were driven by a series genetic pathways. In our study, seven potential candidate genes were identified from five QTL regions according to the reference genome of *D. rerio*, all of them were mapped onto LG25 and LG49 of crucian carp. Among these genes, three candidate genes (*cse1l*, *mapk11* and *myh14*) are molecular functionally related to GTPase or ATP, participating in energy metabolism. Interestingly, two of the candidate genes *cse1l* (chromosome segregation 1-like) and *fam126b* (family with sequence similarity 126, member b) were identified in a 4.2 cM QTL interval of LG25 (qFCE25-b, 21.118–25.275 cM), affecting FCE with the largest PVE of 20.9% and ADG with the PVE of 14.1%, and harboring six SNP markers (ref-112211, ref-36197_17, ref-104702, ref-19027_24, ref-78180_14 and ref-173). *cse1l* has been reported as a nuclear transport factor that plays a critical role in early embryonic development in mice^62^. Furthermore, cse1l also involves in body fluid secretion, and a mutation of cse1l gene in zebrafish leads to sudden and dramatic expansion of the gut tube^63^, indicating that this gene may be one of the major genes affecting the digestion of fish. Another candidate gene, *myh14* (myosin heavy chain 14) was found within the QTL interval of qFCE49-a, which influenced FCE, RGR and ADG with the PVE values of 18.2%, 18.7% and 14.8%, respectively. The expression pattern of *myh14* in zebrafish revealed its species-specific functions in fish muscle formation^64^, implying that this gene may influence the swimming ability of fish. The candidate gene *rgs9bp* is a regulator of G-protein signaling, which involves in bone metabolism in catfish^65^. Whereas, *cldn10a* and *cldn10b* are associated with salinity regulation in Japanese medaka (*Oryzias latipes*)^66^. It is unexpected that no feed efficiency-associated candidate genes were detected in other QTL intervals or nearby regions because the markers within these regions failed to be mapped to current assemblied genome of crucian carp. Additional 3^rd^ generation sequencing (e.g Pacbio) data may improve the genome integrity of crucian carp and help to identify more candidate genes potentially related to feed efficiency. Obviously, genes and markers identified in this work need further validation for their functional relatedness with feed efficiency in the future.

Some candidate genes involved in feed efficiency have also been reported in other animals. Houston *et al*. (2005) found that the significant QTL for feed efficiency lied close to the insulin-like growth factor 2 gene (IGF2) in pigs^67^. Rasal *et al*. (2015) reported that TGF-β receptor type 3 was a candidate gene associating with an extremely significant difference in the FCR in chicken^68^. Compared to common carp, in which eighteen candidate genes were obtained from eight QTL regions affecting FCR^17^, more QTL regions were identified but less genes associating feed efficiency were found in this study. Two factors might cause these differences in two cyprinid fish. First, it is difficult to identify potential genes from the QTL regions for some aquaculture animals as they always have relative large quantities of DNA, in which more non-coding DNA than coding DNA in their genome^17^. Second, some SNP sequences obtained by 2b-rad technology may be difficult to align to a unique region of the crucian carp genome that has replicated internally and undergone the fourth round of whole genome duplication (4R-WGD)^45^.

In conclusion, a high-resolution linkage map of crucian carp was constructed using 8,460 SNPs with an average density of 0.478 cM/marker. Totally, 35 QTL affecting the FCE, RGR, ADG and ADFI were mapped on 14 LGs, which explained 14.0–20.9% of the phenotypic variations. Several QTL influencing different traits of FCE and its close relatives were clustered in identical or close regions of the same linkage group (LG16, LG25, LG36 and LG49). More attentions should be taken to those QTL shared by different traits as they may be controlled by same or similar genomic regions which would be valuable for genetic studies towards the improvement of target traits^69^. Seven candidate genes were identified from five QTL regions in this study and some of these genes are functionally related to energy metabolism, digestion, biosynthesis and signal transduction. Our study provides a basis for elucidating molecular mechanism of feed efficiency, and informative genomic resources for future MAS to the improvement of feed conversion efficiency in crucian carp and its close relatives.

## Materials and Methods

### Ethics statement

All experimental procedures involving the fish in this study were approved by the Committee for Animal Experiments of the Institute of Hydrobiology, the Chinese Academy of Sciences, China. The methods used in this study were carried out in accordance with the Laboratory Animal Management Principles of China.

### Fish and data collection

A large number of wild diploid crucian carp individuals (n = 200) were collected from Zhangdu Lake, Yangtze River (Wuhan, China) as brood fish, and genetic distances among these fish were estimated using a panel of polymorphic microsatellite markers. Then 12 female and 13 male mature fish (generation F_0_) were used to generate 14 families (F1) by artificial crossing in April, 2015. Larval fish of each family were raised in small tanks separately and first fed with Artemia nauplii and then pallet food. At last, 120 fingerlings were randomly selected from one of the families as the fish panel for feed conversion test, whose genetic distance of their dam and sire was the largest among 14 families. This panel was used for genetic linkage map construction and QTL analysis in this study. Fish would snatch food at feeding time when reared in groups as we did in aquaculture practice in ponds or net-cages, while fish would not eat positively in isolated indoor environment at the first beginning. In order to eliminate potential differences as much as possible, we trained the fish of reference family in a concrete indoor tank (about 3 m^3^) and let them be adapted to the fish-feeder’s behaviors for a month before the feed conversion trail. During this month, the fish-feeder clapped hands before each feeding, and then fish gradually snatched food at feeding time until “satiation”. During the feed conversion trail, 120 fish at 82 days post hatch (dph) were reared individually in a series of re-circulating aquarium tanks in order to achieve accurate feed consumption, and fish would still show behaviors of snatching food after the fish-feeder clapped hands as the fish had adapted. All the conditions of the aquariums, such as water temperature (28 °C), dissolved oxygen (7–8 mg/L) and water flow rate (1ms-1), were regularly maintained throughout the experiment. All experimental fish were fed three times (10:00 am, 15:00 pm and 20:00 pm) a day by the same fish-feeder all along the experiment to avoid possible bias of feeder effects. The feeder observed fish carefully during feeding time and stopped feeding when the fish no longer show apparent behavior of snatching food, which was the criterion of “satiation” (about one hour each meal). According to this practice, no feed left in the tanks and therefore no waste of feeds would happen in the experiment. The pallet feeds used in this experiment contain 34.25% crude protein, 9.93% crude lipid and 7.44% ash, which meets aquaculture industry standard of China. The faeces in each tank were siphoned out daily and a complete water change was made every week.

Phenotypic data of the feed conversion efficiency (FCE), relative growth rate (RGR), average daily gain (ADG) and the average daily feed intake (ADFI), were collected after two month feeding trail. Briefly, individual body weight (BW) was recorded at the beginning (initial BW, BW_I_) and the end (final BW, BW_F_) of the feeding test. FCE was calculated as the BW gain after the experiment divided by total feed intake. The RGR was the ratio of the difference between BW_F_ and BW_I_ to BW_I_, while ADG was the ratio of body weight gain to the days of the trail. Total feed intake was recorded as the difference between the final and the beginning weight of diet used during the test, which measured every week, and ADFI was the ratio of the total feed intake to the days of the trail. Curve estimation was used to analyze the correlation between every two traits using SPPSS 13.0 software.

### 2b-RAD sequencing and SNP genotyping

Fin clips of 113 progenies and two parents were sampled and stored in 100% ethanol for DNA extraction using a traditional phenol–chloroform method^70^. The concentration of extracted DNA was measured using a spectrophotometer (Thermo Scientific, USA). 2b-RAD Libraries for all parental and progeny samples were prepared according to the standard protocol^43^ with some modifications. We used 200 ng genomic DNA from each individual as template, and digested them by *Bcg*I restriction enzyme (NEB, UK) at 37 °C for 4 hours. The digestion products were ligated to adaptors (adapter 1 and adapter 2) with 5′- NN - 3′ overhangs at 16 °C overnight. Then the ligation fragments were amplified with Phusion High-Fidelity DNA Polymerase (Thermo Scientific, USA) and a unique 6-bp barcode was used in each library. The amplification products were purified from 10% polyacrylamide gels and recovered using Poly-Gel DNA Extraction Kit (Omega Bio-Tek, USA). Finally, each library for individual progeny was pooled with equal amount in the final mixed library to make the same concentration for each individual, while the parents pooled 3 times the amount of each progeny in the final mixed library to discover segregating SNPs as much as possible. The final library was sequenced by the Illumina HiSeq. 2500 SE50 platform (Illumina, USA) in Anoroad Biotech Inc. (Beijing, China). Low quality reads filering and SNP genotyping followed the procedures previously described by Fu *et al*. (2016)^71^.

### Linkage map and QTL analysis

SNP markers with significant segregation distortion and those could not be genotyped in at least 80% of the progenies were removed^71^. The remaining markers were used for further linkage map construction, which was created by JoinMap 4.1 software^44^ with the regression mapping algorithm. 50 linkage groups (LGs) were constructed at a threshold LOD value of 11.5. The Kosambi mapping function was used to estimate map distances in centiMorgans (cM). Graphical visualization of the linkage map was applied by MapChart 2.2 software. A synteny analysis was done in order to make the LG numbered consistently with a recent crucian carp map^45^.

The multiple QTL mapping (MQM) method was applied to detect any significant associations between marker loci and phenotypic traits in the data sets by MapQTL version 6.0^48^. Cofactors are selected by multiple regression and backward elimination. LOD statistics were calculated at an interval of 1 cM. Permutation tests (10,000 replicates) were utilized to calculate the genome-wide (α < 0.01, n = 1,000) and chromosome-wide (α < 0.05, n = 1,000) LOD score significance thresholds^72,73^ in MapQTL with a confidence interval of 95%. MapChart 2.2 software was used to produce graphic images of QTL profiles at last.

### Identification of potential candidate genes

Because a *Bcg*I 2b-rad read sequence is only 32 bp in length, it is too short to be used in the blast searches for potential genes from public databases. Extending flanking sequences of these SNP markers that located in the confidence intervals of the QTL was performed by adding 300 bp from each side of the 2b-rad sequence in the crucian carp genome (Jingou Tong *et al*., unpublished data). The extended sequences were then used to blast the genome of *D. rerio* (a closely-related species with crucian carp in the same family Cyprinidae, http://www.ensembl.org/Danio_rerio/Info/Index) for identifying potential candidate genes, which may be related to feed efficiency based on the annotation information.

## Electronic supplementary material

Supplementary materials

Supplementary Table S1

Supplementary Table S2
