# Supplementary material for: Quantitative trait loci mapping for feed conversion efficiency in crucian carp (Carassius auratus)
Source: Sci Rep. 2017 Dec 5;7:16971. doi: 10.1038/s41598-017-17269-2 (PMC5717303; doi:10.1038/s41598-017-17269-2)
Supplement: Supplementary file 1 — Supplementary materials [file 41598_2017_17269_MOESM1_ESM.doc]

# Quantitative trait loci mapping for feed conversion efficiency in crucian carp (*Carassius auratus*)

Meixia Pang^1,2^, Beide Fu^1^, Xiaomu Yu^1^, Haiyang Liu^1,2^, Xinhua Wang^1,2^, Zhan Yin^1^, Shouqi Xie^1^, Jingou Tong ^1,*^

^1^State Key Laboratory of Freshwater Ecology and Biotechnology, Institute of Hydrobiology, Chinese Academy of Sciences, Wuhan, 430072, China

^2^ University of Chinese Academy of Sciences, Beijing, 100039, China

Meixia Pang: pang1mei2xia3@163.com

Beide Fu: [fubeide@ihb.ac.cn](mailto:fubeide@ihb.ac.cn)

Xiaomu Yu: [xmyu@ihb.ac.cn](mailto:xmyu@ihb.ac.cn)

Haiyang Liu: 15807125357@163.com

Xinhua Wang: [xinhuawang123@163.com](mailto:xinhuawang123@163.com)

Zhan Yin: zyin@ihb.ac.cn

Shouqi Xie: sqxie@ihb.ac.cn

Jingou Tong: [jgtong@ihb.ac.cn](mailto:jgtong@ihb.ac.cn)

Corresponding author: Jingou Tong

Correspondence and requests for materials should be addressed to J.Tong

Email: [jgtong@ihb.ac.cn](mailto:jgtong@ihb.ac.cn)

Tel: +86 27 68780751

Fax: +86 27 68780123

**Supplementary materials**

**Supplementary Table S1.** Information of 8,460 SNP markers located in the high-density linkage map of crucian carp.

**Supplementary Table S2.**  Detailed information for markers used to blast candidate genes.
